# Multicenter Study of *Cronobacter sakazakii* Infections in Humans, Europe, 2017

**DOI:** 10.3201/eid2503.181652

**Published:** 2019-03

**Authors:** Sarah Lepuschitz, Werner Ruppitsch, Shiva Pekard-Amenitsch, Stephen J. Forsythe, Martin Cormican, Robert L. Mach, Denis Piérard, Franz Allerberger, the EUCRONI Study Group

**Affiliations:** Austrian Agency for Health and Food Safety, Vienna, Austria (S. Lepuschitz, W. Ruppitsch, S. Pekard-Amenitsch, F. Allerberger);; Vienna University of Technology, Vienna (S. Lepuschitz, R.L. Mach);; foodmicrobe.com, Keyworth, United Kingdom (S.J. Forsythe);; National University of Ireland, Galway, Ireland (M. Cormican);; Universitair Ziekenhuis Brussel, Brussels, Belgium (D. Piérard)

**Keywords:** Cronobacter sakazakii, public health surveillance, foodborne diseases, bacteria, bacterial typing, whole-genome sequencing, outbreak investigation, Europe, food safety

## Abstract

*Cronobacter sakazakii* has been documented as a cause of life-threating infections, predominantly in neonates. We conducted a multicenter study to assess the occurrence of *C. sakazakii* across Europe and the extent of clonality for outbreak detection. National coordinators representing 24 countries in Europe were requested to submit all human *C. sakazakii* isolates collected during 2017 to a study center in Austria. Testing at the center included species identification by matrix-assisted laser desorption/ionization time-of-flight mass spectrometry, subtyping by whole-genome sequencing (WGS), and determination of antimicrobial resistance. Eleven countries sent 77 isolates, including 36 isolates from 2017 and 41 historical isolates. Fifty-nine isolates were confirmed as *C. sakazakii* by WGS, highlighting the challenge of correctly identifying *Cronobacter* spp. WGS-based typing revealed high strain diversity, indicating absence of multinational outbreaks in 2017, but identified 4 previously unpublished historical outbreaks. WGS is the recommended method for accurate identification, typing, and detection of this pathogen.

*Cronobacter sakazakii* is a motile, gram-negative, rod-shaped opportunistic pathogen of the family *Enterbacteriaceae* (*1*). In 2007, organisms previously classified as *Enterobacter sakazakii* were reassigned to the new genus *Cronobacter*, which now consists of 7 species: *C. sakazakii*, *C. condimenti*, *C. dublinensis*, *C. malonaticus*, *C. muytjensii*, *C. turicensis*, and *C. universalis* (*2**,**3*). *C. sakazakii* has been isolated from various environments (e.g., domestic environments and manufacturing plants), clinical sources (e.g., cerebrospinal fluid, blood, and sputum), food (e.g., cheese, meat, and vegetables), and animals (e.g., rats and flies) (*4**,**5*).

Most reported cases of illness caused by *C. sakazakii* are in infants <2 months old (*6*,*7*). Premature infants and infants with underlying medical conditions are at the greatest risk for illness. Numerous outbreaks caused by *C. sakazakii* have been traced to contaminated powdered infant formula (*8*). Powdered infant formula is not a sterile product, and the ability of *C. sakazakii* to tolerate dry conditions enables it to survive for long periods in the final powdered product (*9*).

The screening of food (particularly powdered formula) was proposed to reduce the risk to neonatal and infant health (*10*,*11*). The most common syndromes of foodborne infection in infants include necrotizing enterocolitis (NEC), bacteremia, and meningitis (*12*,*13*). Examples of outbreaks of illness in hospital neonatal units caused by *C. sakazakii* associated with powdered infant formula have been compiled by Iversen and Forsythe (*6*) and by Lund (*8*).

A few cases of illness (usually nongastrointestinal) in adults caused by *C. sakazakii* have been reported. In most of these cases the adults had underlying diseases, and no evidence of foodborne transmission was reported (*14*,*15*).

We performed a multicenter study of *C. sakazakii* infections in humans (EUCRONI) to determine the occurrence of *C. sakazakii* in clinical microbiology laboratories across Europe. We also assessed the extent of clonality for human *C. sakazakii* isolates.

## Material and Methods

### Study Design

**Figure 1:**
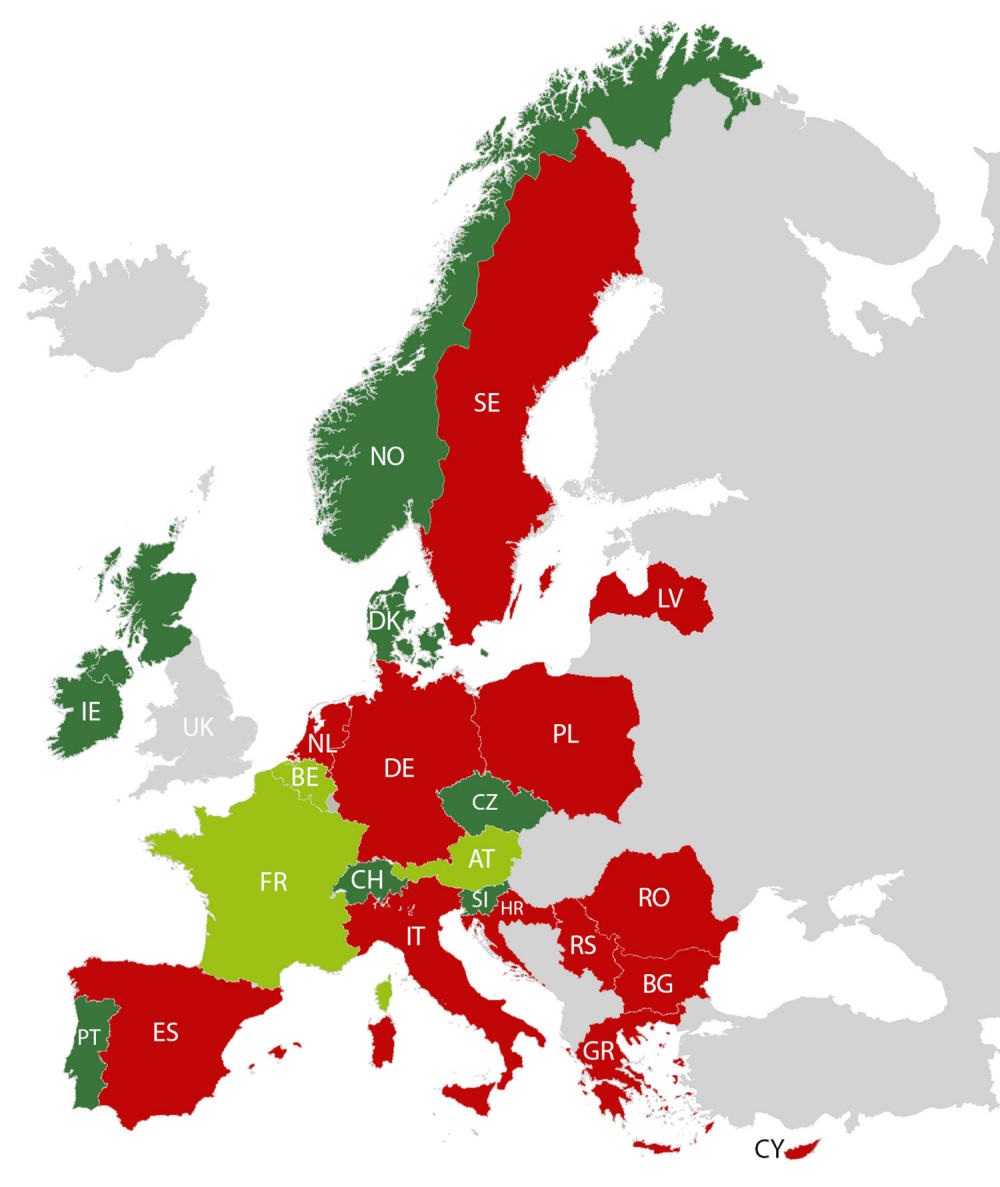
Countries participating in a multicenter study of *Cronobacter sakazakii* infections in humans, Europe, 2017. Dark green indicates the 8 countries that sent *C. sakazakii* isolates to the study center in Austria; light green indicates the 3 countries where historical outbreaks were detected; and red indicates the 13 countries that participated but did not provide isolates. AT, Austria; BE, Belgium; BG, Bulgaria; CH, Switzerland; CY, Cyprus; CZ, Czech Republic; DE, Germany; DK, Denmark; ES, Spain; FR, France; GR, Greece; HR, Croatia; IE, Ireland; IT, Italy; LV, Latvia; NL, Netherlands; NO, Norway; PL, Poland; PT, Portugal; RO, Romania; RS, Serbia; SE, Sweden; SI, Slovenia; UK, United Kingdom.

EUCRONI consisted of national coordinators (EUCRONI study group members) from 24 countries in Europe. Coordinators had to actively approach all medical microbiology laboratories to collect human *C. sakazakii* isolates (1 per patient) in their respective countries during 2017. Human historical isolates (with isolation dates before 2017) were also accepted. The 24 participating countries were arbitrarily chosen to reflect a wide geographic and socioeconomic range (Figure 1). Isolates were transferred to the study center (Austrian Agency for Health and Food Safety, Vienna, Austria) for whole-genome sequencing (WGS), matrix-assisted laser desorption/ionization time-of-flight (MALDI-TOF) mass spectrometry (MS) analysis, and antimicrobial drug susceptibility testing. We submitted data capture forms to national coordinators to collect the following demographic data: patient age and sex, patient status (colonized or infected), specimen source, type of healthcare facility requesting the microbiologic culture, and date of specimen collection.

### Species Identification and DNA Extraction

We cultured isolates on Columbia blood agar plates (bioMérieux, https://www.biomerieux.com/) overnight at 37°C. We performed species identification by using MALDI-TOF Biotyper (Bruker, https://www.bruker.com) and MBT Compass IVD 4.1.60 (Bruker) according to the manufacturer’s instructions. We conducted isolation, quantification, and WGS of genomic DNA according to methods described by Lepuschitz et al. (*16*). We used Sequencing Coverage Calculator (https://www.illumina.com) for calculation of a desired mean coverage of >80-fold.

###   WGS Data Analysis

We de novo assembled raw reads by using SPAdes version 3.9.0 (*17*) and processed them in SeqSphere+ (Ridom GmbH, https://www.ridom.de) for bacterial typing. We deposited the genome sequences in the PubMLST *Cronobacter* database (https://pubmlst.org/Cronobacter) under accession nos. 2403 and 2495–2552. To determine the core genome multilocus sequence type (cgMLST) gene set, we performed a genome-wide gene-by-gene comparison by using the MLST+ target definer function of SeqSphere+ as described previously (*18*) with default parameters and the complete genome of *C. sakazakii* strain ATCC BAA-894 (*19*) as reference genome, all complete *C. sakazakii* genomes available at GenBank, 8 isolates retrieved from whole-genome shotgun sequencing projects, and 4 *C. sakazakii* isolates sequenced at the Austria study center as query genomes. We extracted sequences of the 7 genes comprising the allelic profile of the classical MLST scheme and queried them against the *C. sakazakii* MLST database (*1*), assigning classical sequence types (STs) in silico. We obtained additional species confirmation by using JSpeciesWS (*20*) and ribosomal MLST (*21*). We included 23 *C. sakazakii* historical isolates from 4 different outbreaks (F. Allerberger, 2016; F. Barbut, 2010–2016; G. Feierl, 2009; D. Piérard, 1997–1998, all unpub. data; Appendix Table 1 and 3 reference strains, ATCC BAA-894 (*19*), ATCC29544 (PRJNA224116), and NCTC 8155 (PRJNA224116), to determine the level of microevolution.

### Antimicrobial Resistance Testing

We performed in vitro susceptibility testing with the VITEK 2 Compact System (bioMérieux) and interpreted the VITEK 2 AST196 card according to European Committee on Antimicrobial Susceptibility Testing criteria for *Enterobacteriaceae* (Clinical Breakpoint Tables version 8.0, http://www.eucast.org/ast_of_bacteria/previous_versions_of_documents). For detection of antibiotic resistance genes, we used the Comprehensive Antibiotic Resistance Database (*22*) with default settings “perfect” and “strict” for sequence analysis. We tested isolates in SeqSphere+ for *Cronobacter*-specific variant *ampC* (e.g., CSA-1, CSA-2, CMA-1, and CMA-2) (*23*).

## Results

### Strain Collection and Primary Species Identification

During the study period, 11 of 24 national coordinators (Figure 1) provided 77 presumptive *C. sakazakii* isolates previously identified by conventional biochemical testing, local MALDI-TOF MS analysis (Bruker Biotyper and VITEK MS), locally performed *Cronobacter* genus- and species-specific PCRs, or 16S rRNA gene sequence analysis. These 77 isolates consisted of 36 human isolates from 2017 and 41 historical human isolates obtained during 1964–2016. The participating laboratories, using local conventional phenotypical methods or local MALDI-TOF MS analysis, incorrectly identified 18 (23.4%) of 77 human isolates as *C. sakazakii*. 

MALDI-TOF MS analysis in the study center identified 69 of 77 isolates as *C. sakazakii;* 1 isolate from 2017 yielded low-confidence identification (log[score] value 1.70–1.99). We assigned 7 clinical isolates from 2017 and 1 historical clinical isolate from 2005 to other species (Table 1). The WGS-based species identification using JSpeciesWS and rMLST confirmed MALDI-TOF MS identification results in all but 10 of the 69 isolates. WGS indicated that 5 isolates were *C. malonaticus*, 2 were *C. turicensis*, 1 was *C. dublinensis*, 1 was *C. universalis*, and 1 was *Siccibacter turicensis* (Table 1; Appendix Table 1).

**Table 1:** Comparison of MALDI-TOF mass spectrometry and whole-genome sequencing results for 77 isolates submitted as *Cronobacter sakazakii* in a multicenter study of *C. sakazakii* infections in humans, Europe, 2017*

MALDI-TOF	Whole-genome sequencing	Total no. isolates	Human isolates detected in 2017	Historical human isolates
*C. sakazakii*	*C. sakazakii*	59	21	38
*C. sakazakii*	*C. dublinensis*	1	1	–
*C. sakazakii*	*C. malonaticus*	5	4	1
*C. sakazakii*	*C. turicensis*	2	1	1
*C. sakazakii*	*C. universalis*	1	1	–
*C. sakazakii*	*Siccibacter turicensis*	1	1	–
*Enterobacter aerogenes*	*Kluyvera intermedia*	1	1	–
*E. asburiae*	*E. cloacae*	2	2	–
*E. asburiae*	*E. asburiae*	1	–	1
*Klebsiella oxytoca*	*Klebsiella oxytoca*	1	1	–
*Kosakonia cowanii*	*Kosakonia cowanii*	2	2	–
*Paenibacillus pasadenensis*	*Paenibacillus pasadenensis*	1	1	–

*MALDI-TOF, matrix-assisted laser desorption/ionization time-of-flight; –, no isolates detected.

### Human *C. sakazakii* Isolates Collected in 2017

In total, 21 *C. sakazakii* isolates from 21 patients were collected in 2017 in 9 participating countries in Europe. Case-fatality ratio (within 30 days after specimen collection) was 2 of 21 case-patients (Table 2).

**Table 2:** Characteristics of patients enrolled and *Cronobacter sakazakii* isolates collected in a multicenter study of *C. sakazakii* infections in humans, Europe, 2017*

Sample ID	Country of origin	Patient age, y/sex	Specimen source	Death within 30 d	MLST
802520	Austria	73/F	Stool	No	630
7750-17	Austria	<1/M	Blood	Yes	4
16862-17	Austria	77/F	Blood	No	37
808921	Austria	69/F	Stool	No	21
56487-17	Austria	78/M	Urine	No	17
101807-17	Austria	77/M	Blood	No	1
9929-17	Austria	5/M	Stool	No	17
EUCRONI016	Belgium	61/M	Urine	No	13
EUCRONI012	Belgium	78/M	Wound	No	31
1481-17	Czech Republic	80/F	Rectal swab	No	8
436-17	Czech Republic	31/M	Rectal swab	No	4
10965-17	Czech Republic	74/M	Rectal swab	No	4
D97986	Denmark	85/F	Sputum	No	1
17007483	Denmark	69/M	Urine	No	58
423410	Ireland	65/M	Blood	No	12
170215-0130	Norway	87/M	Blood	Yes	17
M732000	Portugal	60/M	Urine	No	40
80357408-17	Scotland	73/F	Stool	No	33
80363028-17	Scotland	71/M	Urine	No	4
07_2005	Slovenia	54/M	Tracheal aspirate	No	184
2017C1	Switzerland	55/F	Cervix uteri	No	40

*MLST, multilocus sequence type.

### Molecular Typing of Bacterial Isolates

The defined cgMLST gene set consisted of a total of 2,831 core and 1,017 accessory targets. Of 77 sequenced isolates, 59 isolates were confirmed as *C. sakazakii*. These isolates had on average 99.4% of good core genome targets (97.7% to 99.9%) (*18*) and revealed in total 17 different sequence types (STs) (Table 3).

**Table 3:** In silico evaluation of MLSTs for *Cronobacter sakazakii* strains in a multicenter study of *C. sakazakii* infections in humans, Europe, 2017*

MLST	Total no. isolates	Human isolates detected in 2017	Historical human isolates
1	7	2	5
12	1	1	–
13	1	1	–
148	1	–	1
155	10	–	10
17	3	3	–
184	1	1	–
21	10	1	9
31	2	1	1
33	1	1	–
37	1	1	–
4	11	4	7
40	2	2	–
50	1	–	1
58	1	1	–
630	1	1	–
8	5	1	4

*MLST, multilocus sequence type; –, no isolates detected.

**Figure 2:**
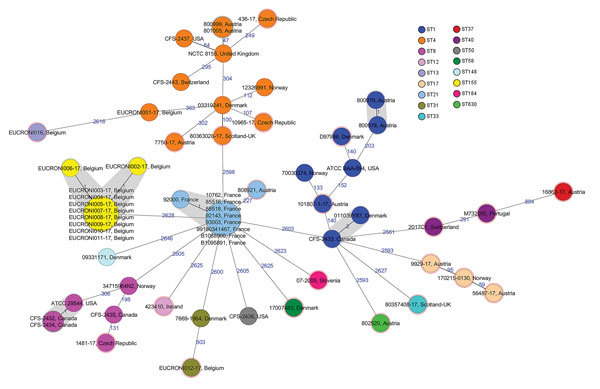
Minimum-spanning tree of 59 *Cronobacter sakazakii* isolates, including 21 human isolates from 2017 and 38 historical human isolates, from 11 countries in Europe. Each circle represents isolates with an allelic profile based on the core genome multilocus sequence type, which consists of 2,831 alleles. Blue numbers indicate the allelic differences between isolates; isolates with closely related genotypes are shaded in gray. Isolates were colored according to classical multilocus sequence type, labeled with the country of isolation and the respective sample identification. Nodes encircled with a dotted red line were collected in 2017. Ireland additionally provided 7 historical isolates originating from Canada (n = 4), United States (n = 2), and Switzerland (n = 1). For comparison, sequence data of reference strains ATCC BAA-894 (United States, ST1), ATCC29544 (United States, ST8), NCTC 8155 (United Kingdom, ST4) were included. ST, sequence type.

Core genome comparison of 59 *C. sakazakii* isolates and the 3 reference strains revealed an average allelic difference of 2,402 and a maximum allelic difference of 2,724 (Figure 2). Isolates clustered in the minimum-spanning tree to their respective MLST. Eight isolates belonging to ST1 included 2 stool isolates from neonates with a common epidemiologic link in Austria in 2009; these 8 isolates showed 1 allelic difference and were most closely related (203 alleles difference) to reference ATCC BAA-894, an isolate collected from powdered formula in the United States in 2001. That outbreak affected 2 neonates with necrotizing enterocolitis (both male, age 10 days and 12 days) hospitalized in the same neonatal intensive care unit.

Twelve isolates belonged to ST4, of which 3 were confirmed isolates from infants. Two infant isolates belonged to an outbreak cluster with a common epidemiologic link detected in Austria in 2016; these isolates shared the same cgMLST profile and showed a maximum of 47 allelic differences to the historical reference strain NCTC 8155 (from milk, United Kingdom, 1950). This outbreak again affected 2 neonates (neonate A: female, age 22 days, positive blood culture, fatal outcome; neonate B: male, age 16 days, positive respiratory tract specimen) hospitalized in the neonatal intensive care unit of another hospital in Austria. The third infant isolate was a 2017 ST4 isolate from a case in Austria with a fatal outcome and was most closely related (302 allelic differences) to a historical strain from Denmark isolated in 2003.

Six clinical isolates assigned to ST8 consisted of 2 historical human isolates from Canada (date of isolation unknown). These 6 isolates shared the identical core genome profile and had 1 allelic difference to reference strain ATCC29544 (from an infant, United States, 1970).

Nine human isolates assigned to ST21 comprised a historical outbreak cluster from France collected during 2010–2016. The outbreak included 3 female patients (mean age 62 years) and 5 male patients (mean age 68 years); initial specimens were abscess material from the digestive tract (n = 1), ascites fluid (n = 1), respiratory tract specimens (n = 2), and rectal swab specimens (n = 4). Eight of these 9 isolates showed the same core genome genes, and 1 yielded 1 allelic difference.

All 10 isolates assigned to ST155 belonged to a historical outbreak among infants in Belgium during 1997–1998; the isolates originated from blood cultures (n = 2), stool specimens (n = 2), rectal swab specimens (n = 4), and respiratory tract specimens (n = 2). The first positive sample was collected in November 1997; the remaining 9 specimens were obtained during August–September 1998. Eight of the 10 isolates shared the same cgMLST profile, and 2 had 1 allelic difference.

In total, 27 of 38 historical isolates were most closely related (<1 allelic difference) to other historical isolates; 11 were singletons. All 21 isolates collected in 2017 were singletons, and no close relatedness was evident (>100 allelic differences) between historical isolates and isolates from 2017.

### In Vitro and In Silico Antimicrobial Resistance Analysis

In vitro susceptibility testing of 21 human *C. sakazakii* isolates from 2017 revealed 20 *C. sakazakii* isolates that were susceptible to all 14 tested antibiotics (Appendix Table 2). One isolate was resistant to ampicillin, cefotaxime, gentamicin (intermediate), moxifloxacin, and trimethoprim/sulfamethoxazole.

Of 21 *C. sakazakii* isolates, 12 isolates carried the efflux genes *emrB*, *msbA*, *patA*, regulatory systems modulating antibiotic efflux *CRP*, *marA*, *emrR*, *marR*, H-NS, antibiotic target protection gene *msrB*, and the determinant of fosfomycin resistance *glpT.* Seven isolates had in addition the antibiotic protection gene *vgaC*. One isolate, had also the efflux gene *norB*, the antibiotic inactivation gene *fosX*, and the antibiotic target alteration gene *mprF*. One isolate had the additional antibiotic inactivation genes *aac(6')-Ib-cr*, *aadA16*, *aadA2*, *ant(2'')-Ia*, *arr-3*, *catB3*, *CTX-M-9*, *OXA-1*, the antibiotic target protection gene *qnrA1*, and the antibiotic target replacement gene *sul1*.

The presence of variant *ampC* was confirmed for all 21 isolates. Seventeen isolates harbored CSA-2, and 4 isolates harbored CSA-1 (Appendix Table 2).

## Discussion

The aim of our 2017 *C. sakazakii* study was to assess the occurrence of this opportunistic pathogen in countries of Europe, characterize the isolates, and recognize possible multinational outbreaks. Our finding that only 59 of 77 presumptive *C. sakazakii* isolates had the species-identification *C. sakazakii* confirmed at the central study center shows that correct identification of *Cronobacter* spp. is still a challenge for many routine laboratories.

The prevalence of reported *C. sakazakii* cases was low, with only 11 (45.8%) of 24 participating countries submitting *C. sakazakii* isolates. Clinical isolates from 2017 showed high genetic diversity, indicating that neither multinational nor national outbreaks occurred in 2017 in the 24 countries studied. However, characterization of the historical isolates obtained during this study confirmed occurrence of 4 previously unpublished historical outbreaks: 2 outbreaks from 2009 and 2016 in Austria, 1 from Belgium during 1997–1998, and 1 from France during 2010–2016. Hospitals affected by nosocomial *C. sakazakii* outbreaks might still be reluctant to publish possibly food-related outbreaks or nosocomial infections, especially in the case of affected infants and particularly in the case of related fatalities.

Strain typing using classical MLST identified a total of 17 STs among 59 sequenced *C. sakazakii* isolates. Our addition of a new ad hoc cgMLST scheme consisting of 2,831 core target genes provides more discriminative power for outbreak investigation and source tracking than the standard 7-loci MLST scheme.

The dominant STs found among our clinical *C. sakazakii* isolates from 2017 were ST4, ST17, ST1, and ST40, a distribution consistent with results from other studies (*1*). The medical literature often links *C. sakazakii* ST4 with powdered infant formula–associated outbreaks in infants (*3*). In our study, the sole strain (7750-17) affecting an infant (a 3-month-old baby boy who died) was ST4, isolated from a blood culture.

Antibiotic treatment is essential in the care of a patient with a confirmed *Cronobacter* infection. The traditional antibiotic regimen for *Cronobacter* spp. was ampicillin in combination with either gentamicin or chloramphenicol. In view of claimed resistance to ampicillin and most first- and second-generation cephalosporins, it has been suggested that carbapenems or third-generation cephalosporins be used with an aminoglycoside or trimethoprim/sulfamethoxazole (*24*). In our study, antimicrobial resistance testing showed susceptibility to all tested antibiotics for 20 of 21 human isolates from 2017. In comparison to other members of the family *Enterobacteriaceae*, *Cronobacter* strains seem to be more susceptible against so-called “key access antibiotics” of the World Health Organization’s Model List of Essential Medicines (*25*), such as ampicillin, aminoglycosides, chloramphenicol, and third-generation cephalosporins (the last is included in the List of Essential Medicines only for specific, limited indications) (*26*). For all isolates, we confirmed the presence of 1 of 4 tested *ampC* β-lactamase variants, which confer phenotypic resistance exclusively to first-generation cephalosporins (e.g., cephalothin) but not to ampicillin (*23*). A few studies have reported *Cronobacter* isolates conferring multidrug resistance (*26*), a phenomenon observed in our study only for 1 strain from Slovenia.

Correct species identification within the *Cronobacter* group was a major challenge for 7 of 11 participating laboratories. This identification problem is consistent with numerous misidentifications reported in the literature (*27*,*28*). The discrepancies in correct *Cronobacter* spp. identification on a genus and species level between the study center in Austria and the primary testing laboratories using MALDI-TOF MS is probably attributable to outdated databases used by primary testing laboratories. Nevertheless, our study showed that the overall MALDI-TOF MS performance for *Cronobacter* spp. identification on species level is insufficient and misleading. The databases contained data for *C. sakazakii* only, and therefore all 7 species of the genus *Cronobacter* were identified as *C. sakazakii*. In addition, although a database comment indicated that *Cronobacter* could only be identified on the genus level, the MALDI-TOF MS result simulated the highest identification score for *C. sakazakii*. This shortfall should be corrected by an update of the MALDI-TOF MS databases to enable accurate *Cronobacter* identification at the species level. In comparison, WGS-based species identification represents a major improvement to conventional identification methods and MALDI-TOF MS (*29*). Therefore, we recommend the use of WGS-based identification tools and databases for identification of species within the *Cronobacter* group. 

Adults were the main affected age group in our study. All but 2 of the isolates from 2017 originated from adults. This finding confirms the results from previous recent studies (*14*,*30*) and contradicts statements in numerous medical textbooks, postulating that infants are more often affected than adults (*8*,*31*–*33*).

Our study has some limitations. Lack of information (e.g., detailed epidemiologic and clinical patient data) and misidentification on genus and species levels might have played a role in underestimating the real prevalence rate; 13 of the 24 participating countries did not find or did not submit *C. sakazakii* isolates.

In conclusion, this *C. sakazakii* study in Europe revealed a high strain diversity, which points to highly diverse infection sources and an absence of national or multinational outbreaks in 2017. Correct identification of *C. sakazakii* still poses a diagnostic challenge to many laboratories, and the use of such imperfect detection systems might explain the low prevalence of reported clinical *C. sakazakii* isolates found in this study. WGS data must be used for accurate species identification and high-resolution strain typing. We recommend the inclusion of *C. sakazakii* as a notifiable organism by public health authorities.

Appendix Additional information regarding *Cronobacter sakazakii* in clinical samples submitted by countries participating in a multicenter study of *C. sakazakii* infections in humans, Europe, 2017.
